# Feline immunodeficiency virus (FIV) infection in domestic pet cats in Australia and New Zealand: Guidelines for diagnosis, prevention and management

**DOI:** 10.1111/avj.13166

**Published:** 2022-05-16

**Authors:** ME Westman, SJ Coggins, M van Dorsselaer, JM Norris, RA Squires, M Thompson, R Malik

**Affiliations:** ^1^ Sydney School of Veterinary Science The University of Sydney Sydney New South Wales Australia; ^2^ The Cat Clinic New Town Tasmania Australia; ^3^ The Sydney Institute for Infectious Diseases The University of Sydney Sydney New South Wales Australia; ^4^ College of Public Health, Medical & Veterinary Sciences James Cook University Townsville Queensland Australia; ^5^ Centre for Veterinary Education The University of Sydney Sydney New South Wales Australia; ^6^ School of Veterinary and Animal Science Charles Sturt University Wagga Wagga New South Wales Australia

**Keywords:** antibodies, diagnosis, feline immunodeficiency virus, infection, PCR, review, saliva, treatment, vaccination, veterinary science

## Abstract

Despite the passage of over 30 years since its discovery, the importance of feline immunodeficiency virus (FIV) on the health and longevity of infected domestic cats is hotly debated amongst feline experts. Notwithstanding the absence of good quality information, Australian and New Zealand (NZ) veterinarians should aim to minimise the exposure of cats to FIV. The most reliable way to achieve this goal is to recommend that all pet cats are kept exclusively indoors, or with secure outdoor access (e.g., cat enclosures, secure gardens), with FIV testing of any in‐contact cats. All animal holding facilities should aim to individually house adult cats to limit the spread of FIV infection in groups of animals that are stressed and do not have established social hierarchies. Point‐of‐care (PoC) FIV antibody tests are available in Australia and NZ that can distinguish FIV‐infected and uninfected FIV‐vaccinated cats (Witness™ and Anigen Rapid™). Although testing of whole blood, serum or plasma remains the gold standard for FIV diagnosis, PoC testing using saliva may offer a welfare‐friendly alternative in the future. PCR testing to detect FIV infection is not recommended as a screening procedure since a negative PCR result does not rule out FIV infection and is only recommended in specific scenarios. Australia and NZ are two of three countries where a dual subtype FIV vaccine (Fel‐O‐Vax® FIV) is available and offers a further avenue for disease prevention. Since FIV vaccination only has a reported field effectiveness of 56% in Australia, and possibly lower in NZ, FIV‐vaccinated cats should undergo annual FIV testing prior to annual FIV re‐vaccination using a suitable PoC kit to check infection has not occurred in the preceding year. With FIV‐infected cats, clinicians should strive to be even more thorough than usual at detecting early signs of disease. The most effective way to enhance the quality of life and life expectancy of FIV‐infected cats is to optimise basic husbandry and to treat any concurrent conditions early in the disease course. Currently, no available drugs are registered for the treatment of FIV infection. Critically, the euthanasia of healthy FIV‐infected cats, and sick FIV‐infected cats without appropriate clinical investigations, should not occur.

Feline immunodeficiency virus (FIV) is a lentivirus with many similarities to human immunodeficiency virus (HIV). A critical difference between FIV‐associated disease in cats and HIV‐associated disease in humans stems from the length of time that cats have lived with FIV. While humans are believed to have first been infected with HIV a little over 100 years ago, cats and FIV have co‐evolved for 10–20,000 years, such that viral virulence has been reduced substantially over time.^1^

Seven subtypes (or clades) of FIV (A, B, C, D, E, F and U‐NZenv) have been identified to date based on nucleotide sequence differences.^2^, ^3^, ^4^, ^5^, ^6^, ^7^ These differences may impact disease associations, virulence and protection offered by vaccination.^8^ FIV‐A is predominant in Australia, while FIV‐A and FIV‐C are the most common subtypes reported in New Zealand (NZ). Recombinant subtype viruses exist (e.g., A/B, A/C, etc.) and have been reported in the field.^6^, ^7^, ^9^, ^10^, ^11^

FIV transmission primarily occurs via bite wounds that introduce saliva containing virus and FIV‐infected white blood cells.^12^ Therefore, male cats, especially sexually intact male cats (“toms”), have the highest prevalence of FIV infection.^13^, ^14^, ^15^, ^16^ Indeed, the overall prevalence of FIV in a given environment depends on the density of free‐roaming tom cats.^14^, ^17^ Infection can also occur iatrogenically through inoculation with infected blood or saliva, such as via blood transfusions, inadequate sterilisation of dental and surgical equipment, and by breaches in aseptic technique when using multi‐dose vials.^18^, ^19^ Kittens can also acquire FIV vertically from their mothers, either prenatally (i.e., *in utero*) or postnatally (through contaminated breast milk and/or vaginal fluid),^20^, ^21^ although anecdotally, this seems a less important route of transmission in Australia and NZ.

Australia and NZ, like nearby Southeast Asian countries Japan, Thailand and Malaysia, have a relatively high prevalence of FIV infection compared to most countries, possibly because many owners permit cats to live outdoors and populations of feral cats persist in many locations^13^, ^14^, ^22^, ^23^, ^24^, ^25^, ^26^, ^27^, ^28^, ^29^ (Figure 1). One survey revealed that 83% of pet cats in Australia and NZ currently have, or have had, some level of access to the outdoors.^36^ The prevalence of FIV infection has also been reported to be higher in areas of socioeconomic disadvantage in Australia,^37^ possibly linked to unowned cat populations acting as reservoirs of FIV infection as well as variations in the level of investment in animal control in different jurisdictions.^38^ As a generalisation, FIV is uncommon in well‐run catteries in Australia and NZ,^14^ although endemic FIV can emerge in poorly run catteries or shelters.

**Figure 1:**
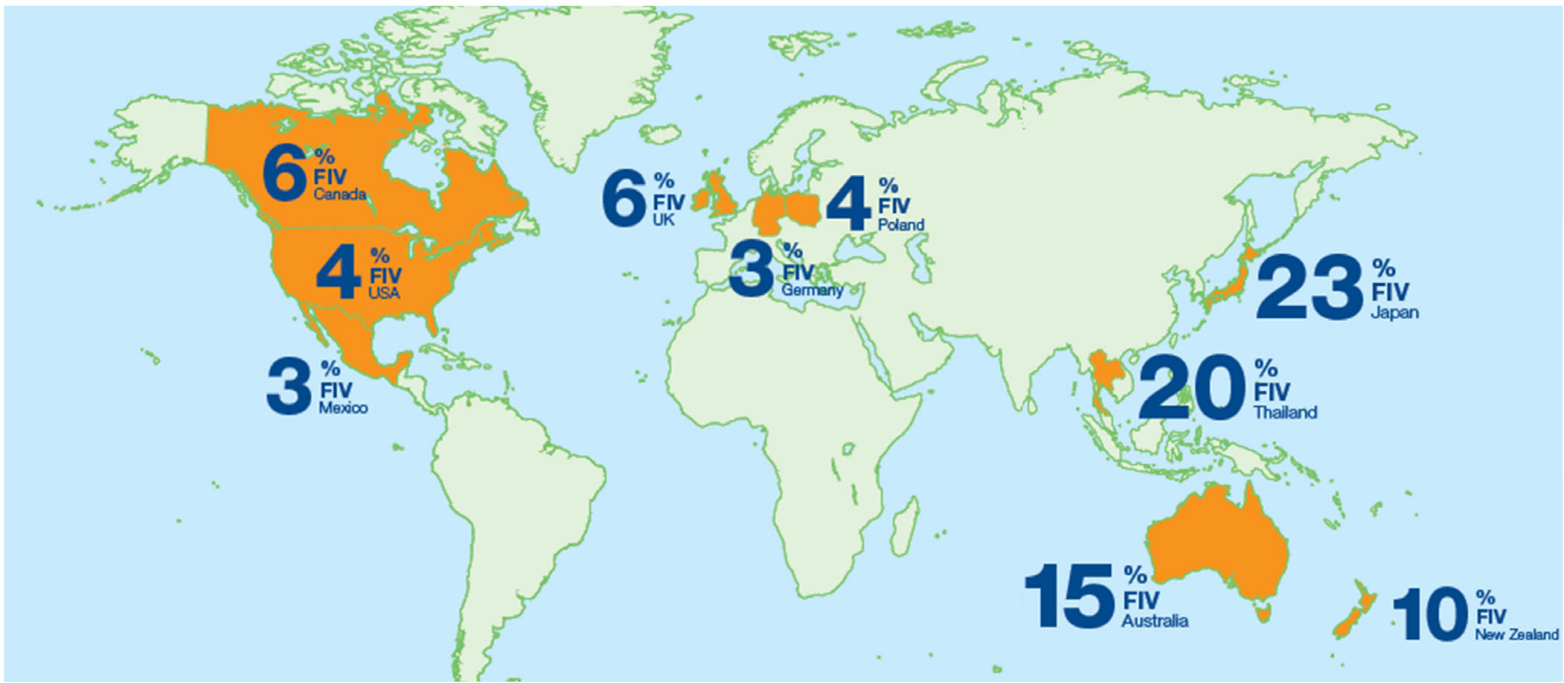
FIV prevalence worldwide. Numbers displayed on the map relate to references 13, 23, 26, 28, 30, 31, 32, 33, 34, 35.

## Pathogenesis (phases of FIV infection)

**Figure 2:**
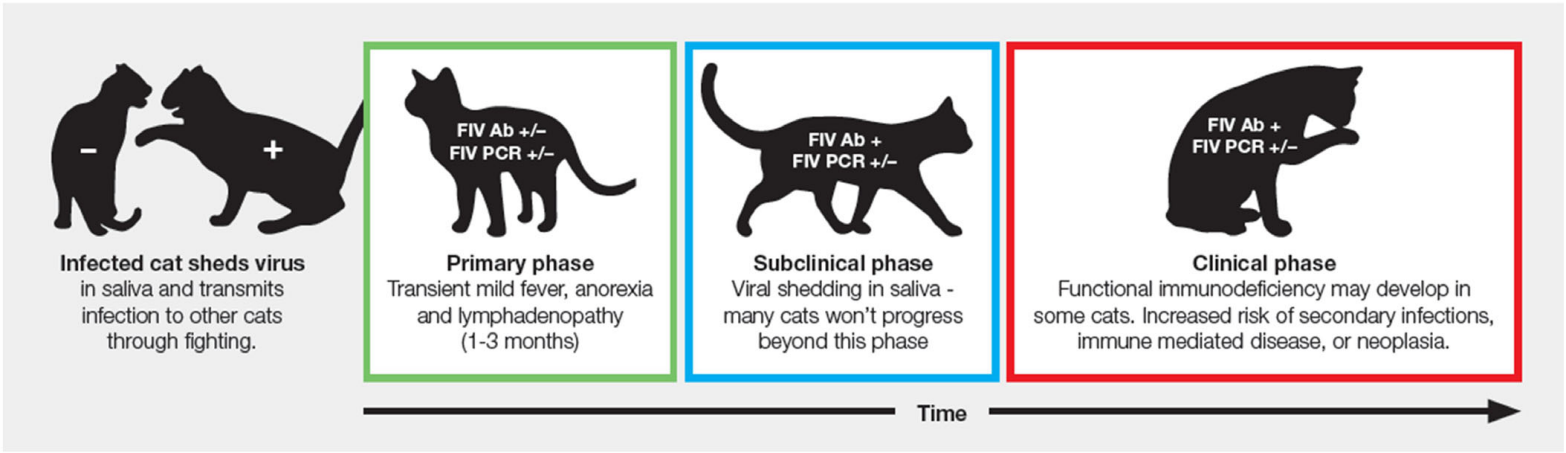
The three phases of FIV infection in cats: Primary, subclinical and clinical. Adapted from the 2020 American Association of Feline Practitioners (AAFP) feline retrovirus testing and management guidelines.^19^ Ab = antibodies, PCR = polymerase chain reaction, + = positive, − = negative.

Three main phases of FIV infection are generally recognised: primary (acute), subclinical and clinical (Figure 2).^19^

**Figure 3:**
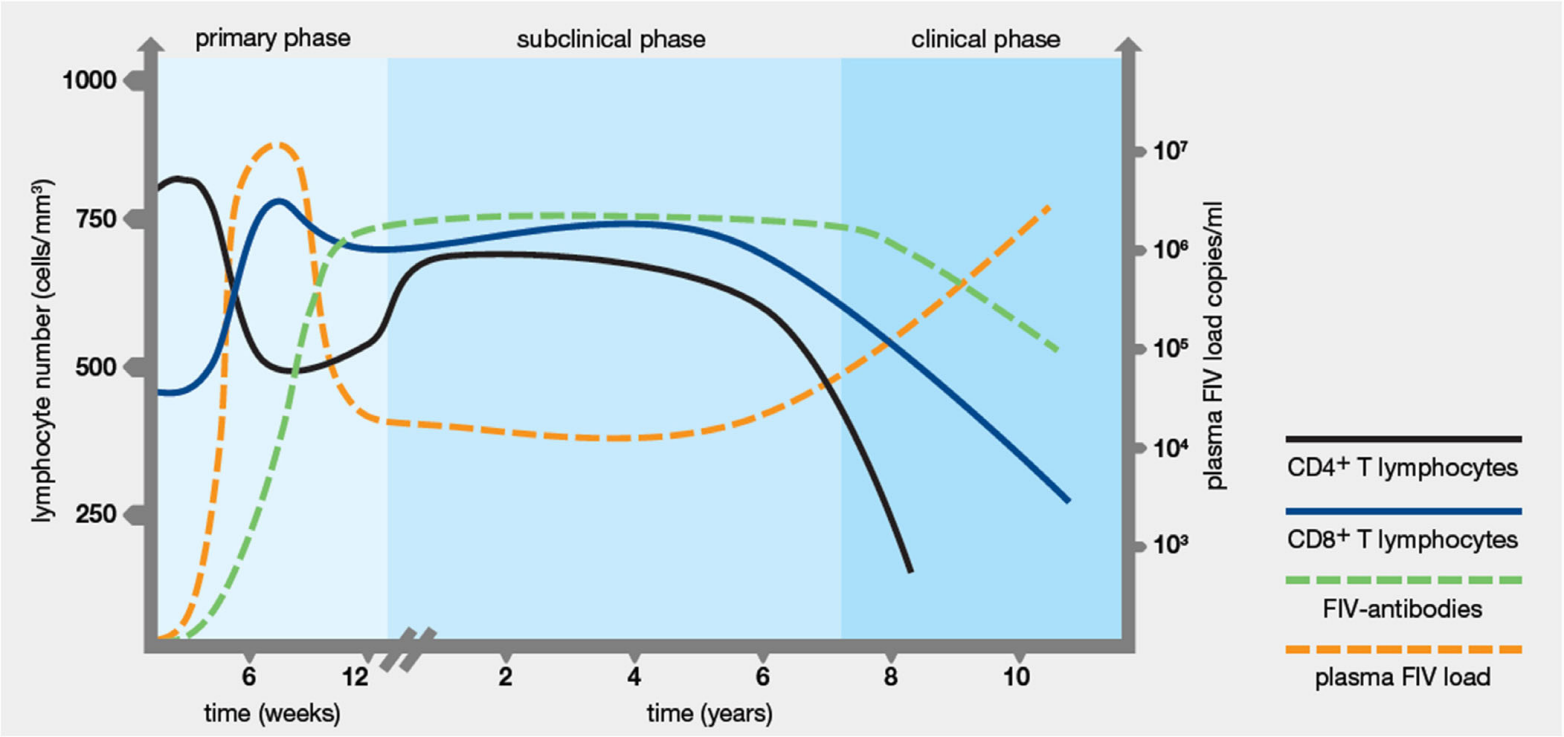
Changes in the levels of FIV antibody, virus, CD4+ T lymphocytes and CD8+ T lymphocytes during the different phases of FIV infection in cats. The *x*‐axis shows the timescale in weeks for the primary phase, and in years for the subclinical and clinical phases. The left *y*‐axis shows CD4+ and CD8+ T lymphocyte numbers (cells/mm^3^). The right *y*‐axis shows the plasma viral load. Figure kindly supplied by Dr Navapon Techakriengkrai, Department of Veterinary Microbiology, Faculty of Veterinary Science, Chulalongkorn University, Thailand.

Following experimental inoculation, FIV infection is associated with transient fever, lymphadenomegaly and lymphopenia, known as the primary phase of FIV infection (1–3 months).^39^, ^40^, ^41^ During this acute phase, FIV can usually be detected in blood with polymerase chain reaction (PCR) testing from two weeks post‐infection, and anti‐FIV antibodies detected from four weeks post‐infection.^19^, ^41^, ^42^ Uncommonly, seroconversion can be delayed for two months or even longer in some FIV‐infected cats.^43^, ^44^ A sharp decline in lymphocyte populations, particularly CD4+ (helper) T lymphocytes, also occurs in the primary phase of infection (Figure 3).^45^, ^46^

Following the primary phase of FIV disease, cats enter a long subclinical phase that can last for many years. During the subclinical phase the production of FIV antibodies is persistently high, and the level of circulating free virus is suppressed, resulting in an undetectably low viral load in some cases (PCR false‐negatives; see later). CD8+ (cytotoxic) T lymphocyte levels increase, which, combined with the dropping level of CD4+ T lymphocytes, produces an inversion of the CD4+/CD8+ ratio early in the subclinical phase. This inversion of the CD4+/CD8+ ratio may persist for life, often reducing from about 3.0 to around 1.0 or less, and can sometimes be useful in diagnosing and staging FIV disease (Figure 3).^42^, ^45^, ^47^, ^48^, ^49^

**Figure 4:**
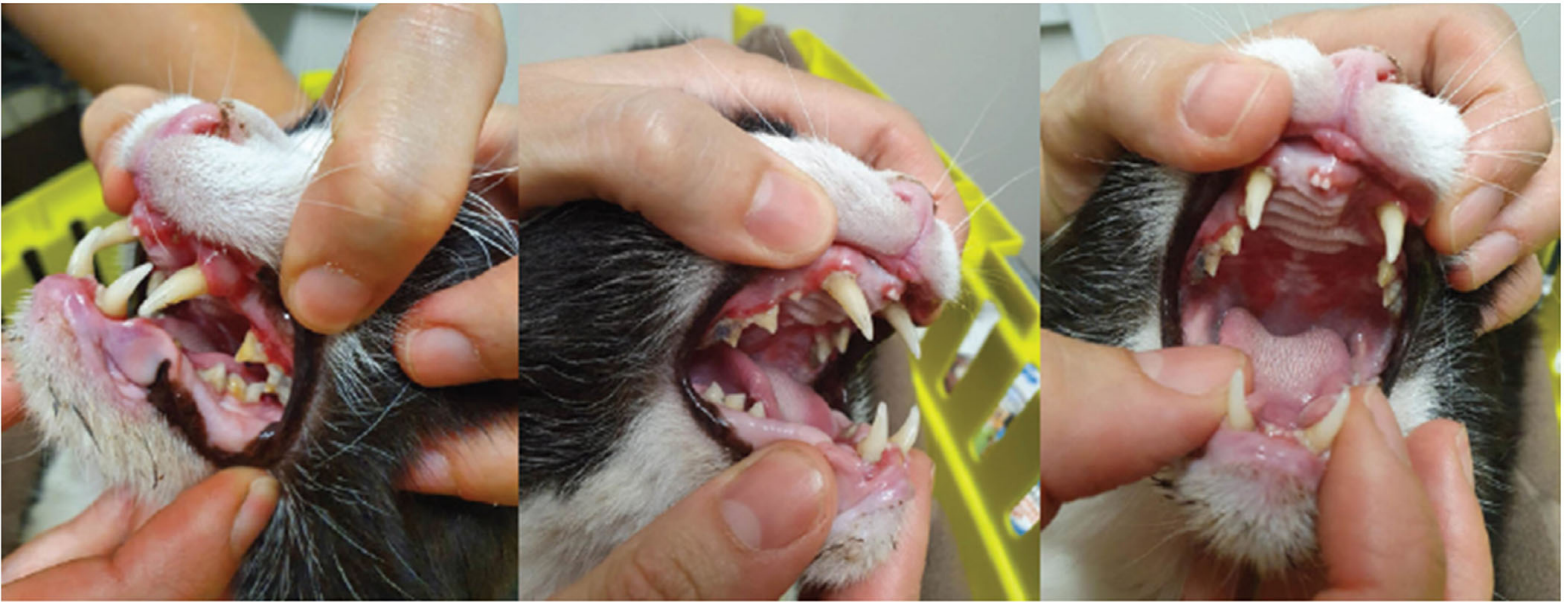
Gingivostomatitis is a common finding in FIV‐infected cats. Images kindly provided by Dr Tessa Clark.

Over time, the general trend with FIV infection is that both CD4+ and CD8+ T lymphocyte numbers gradually decline, causing progressive dysfunction of the immune system until cats enter the clinical phase of FIV infection. During this third phase, FIV‐infected cats are predisposed to chronic and recurrent infections of various types.^19^, ^41^, ^50^ Gingivostomatitis is often present and is classically more severe and refractory to treatment than in FIV‐uninfected cats, and oral resorptive lesions are more common in FIV‐infected cats (Figure 4).^39^, ^45^, ^51^

In people with HIV infection, the marked decline in CD4+ cell numbers results in profound immunodeficiency and the development of opportunistic infections, often sequentially. Although chronic inflammatory conditions and secondary infections can occur in cats with low CD4+ cell counts, the classic opportunistic infections seen in HIV patients (cryptococcosis, *Pneumocystis* pneumonia and *Mycobacterium avium* complex infections) are rarely diagnosed in FIV‐infected cats. Many cats appear to be able to cope with CD4+ cytopenia and remain free of serious infections.^19^

Instead, the most important long‐term impact of FIV infection is the development of neoplasia, typically B cell lymphoblastic lymphoma in the intestine, abdominal lymph nodes or kidneys, although lymphomagenesis usually does not occur until many years after the primary phase of FIV infection.^52^, ^53^, ^54^ Leukaemia/lymphoma is about 6 times more likely in FIV‐infected cats than in FIV‐uninfected cats.^55^ In Australia, one study found 50% (50/101) of cats with naturally‐occurring lymphoma were FIV‐positive, of which 70% (35/50) were B cell phenotype and 50% (25/50) had alimentary lymphosarcoma.^56^

Cellular immunity (mediated by T lymphocytes) is more profoundly affected by longstanding FIV infection than humoral immunity (antibody‐mediated, produced by B lymphocytes).^19^ Hyperglobulinaemia, reflecting nonspecific stimulation of humoral immunity, is commonly reported in FIV‐infected cats.^57^, ^58^, ^59^, ^60^, ^61^ Survival time of FIV‐infected cats is highly variable; while our current understanding is that FIV infection in some cats can have devastating health impacts, in some studies the lifespan of naturally FIV‐infected cats has been similar to that of FIV‐uninfected cats.^30^, ^62^, ^63^, ^64^ This is a confusing concept to convey to colleagues and owners. Many factors including FIV subtype, co‐infections, feline genomics and stress likely impact on disease development (or lack thereof) in FIV‐infected individuals.^50^, ^65^

Studies on the impact of FIV disease rarely document the date of initial infection, nor the subtype involved, so unravelling the actual impact of FIV infection from retrospective data sets is problematic. Therefore, it is often not clear if cats with FIV infection have had the infection for a short time, a moderate period, or for many years – and grouping such cats into the single category of “FIV‐infected” and drawing broad conclusions is misleading. Further problems facing veterinarians are that prospective studies of natural FIV infection are exceedingly rare, and experimental FIV infection studies in laboratory cats usually only last for 6–7 years, and therefore are not a true reflection of what happens in nature.

One study in the USA found that many FIV‐infected cats housed in a high‐stress, large multi‐cat household died of diseases consistent with severe immunodeficiency, most commonly lymphoma. Conversely, in the same study, FIV‐infected cats housed in small, low‐stress groups mostly remained healthy for the duration of the 22‐month study.^65^ These results emphasised the importance of management and housing conditions on the outcomes of FIV infection.

FIV infection is not a death sentence and never constitutes grounds for euthanasia.^19^, ^41^

## Diagnosis of FIV infection

Determining an individual cat's FIV status is useful in assisting with patient management and in managing the risk of FIV transmission to uninfected cats.^19^ FIV testing should be prioritised in cats with sequential opportunistic infections, unexplained neutropenia, anaemia, or lymphoma. Testing with a rapid and affordable FIV point‐of‐care (PoC) kit to detect antibodies against FIV is usually the first step when diagnosing FIV infection. No FIV PoC test is perfect (i.e., 100% sensitive and 100% specific), therefore follow‐up (“confirmatory”) FIV testing is recommended for all FIV‐positive test results, since a diagnosis of FIV infection may result in management changes and/or euthanasia in a shelter setting. Negative FIV test results are generally reliable when highly sensitive PoC tests are used, especially in apparently healthy cats with a low‐risk lifestyle, unless very recently infected.^19^, ^66^

Common sense should be applied to all other testing scenarios by the consulting veterinarian. If FIV testing is unnecessary, and will not change the treatment options, management plan or outcome for a cat, it should not be performed. For example, a 6‐month‐old kitten, born to a known FIV‐uninfected queen, and housed 100% indoors, does not need to be FIV tested prior to rehoming or commencement of FIV vaccination. However, a 6‐month‐old kitten born to a queen of unknown FIV status, or that has had some level of unsupervised outdoor access in the first 6 months of life, should be FIV tested prior to FIV vaccination or co‐mingling with other cats. Similarly, a cat that has been kept 100% indoors since a previous FIV test does not need to be re‐tested for FIV prior to a dental procedure or annual FIV vaccination.

**Figure 5:**
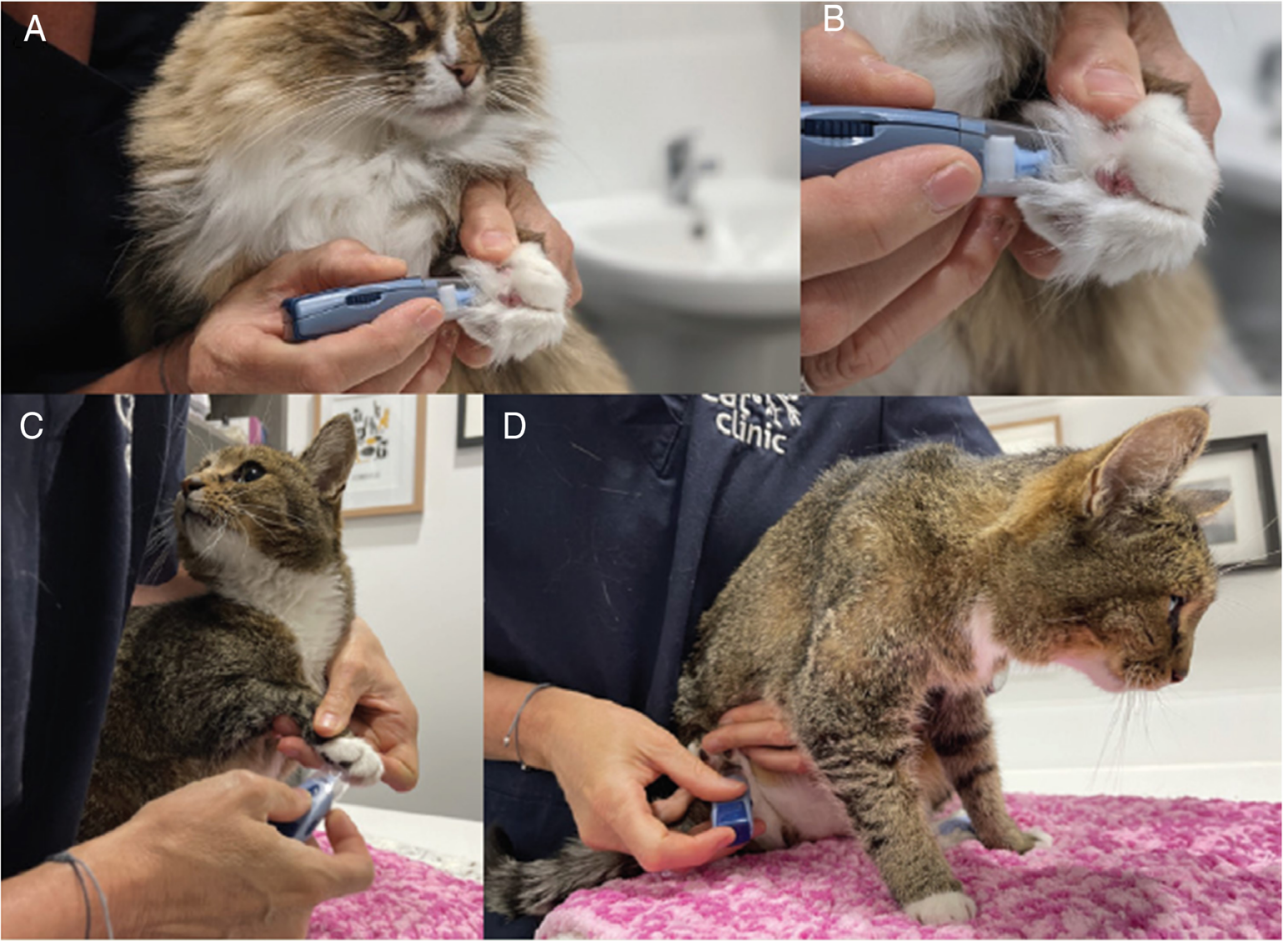
Suggested procedure for foot pad bleeding and FIV point‐of‐care (PoC) testing. The patient is gently restrained in whatever way is most comfortable for the cat. Both metacarpal (A–C) and metatarsal foot pads (D) can be used, depending on which is less stressful for the cat. A lancet device is used to obtain a capillary blood sample by puncturing the skin of the foot pad. Most lancet devices allow for selection of the depth of penetration, and in the case of foot pad collection a slightly deeper penetration is usually required. After lancing, the foot pad is squeezed gently to produce a drop of blood that is placed directly onto the PoC test strip. The test is then performed as per the manufacturer's instructions, using the recommended amount of buffer. Distracting the cat during sample collection with patting or treats is usually all that is required, with most cats barely aware of the sample being taken. Images provided by Dr Moira van Dorsselaer.

**Figure 6:**
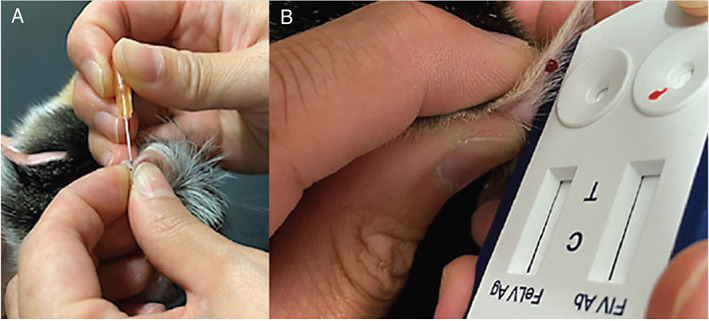
Suggested procedure for ear tip bleeding and FIV point‐of‐care (PoC) testing, adapted from ear prick sampling for blood glucose testing. Ideally, a vein on the dorsal (outside) surface of the pinna is used for sampling; alternatively, if a vein is unable to be visualised (e.g., dark‐coloured cats), the medial (inside) surface of the pinna can be used (A). The pinna should be massaged prior to sampling to encourage blood flow to the tip of the pinna. The skin is pricked with a 25 g needle, and a drop of blood squeezed directly onto the test kit strip (B). Gentle compression of the puncture site afterwards will result in the bleeding stopping. Fractious cats can be gently restrained using a towel wrap while sampling is performed. Images kindly provided by Dr Jeffrey So.

**Figure 7:**
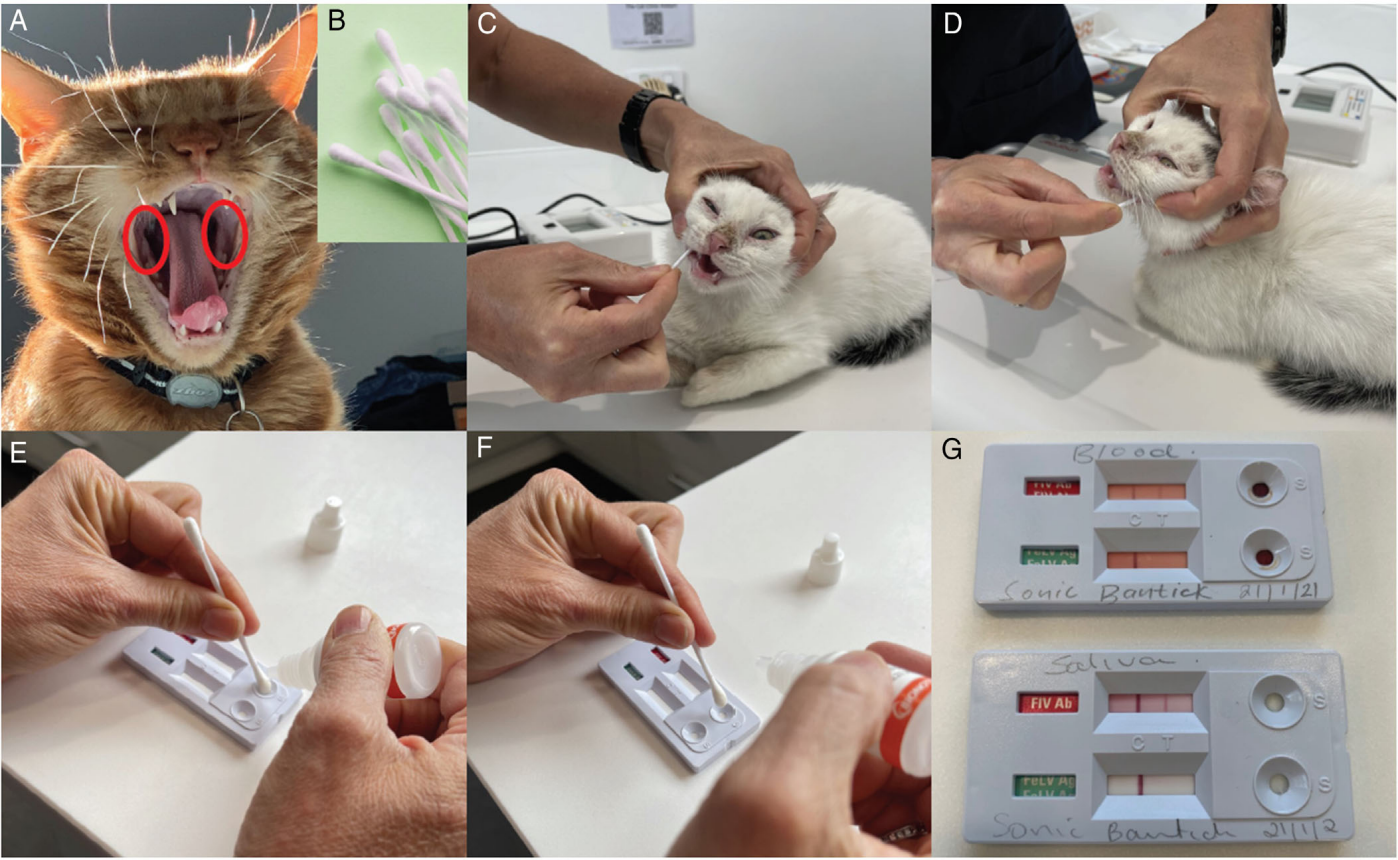
Suggested procedure for saliva testing and FIV point‐of‐care (PoC) testing. Saliva is collected from the caudal oral mucosa (A) (circled in red) using a clean cotton tip (B). The patient is held gently around the head so that the swab can be moved around freely to collect saliva (C) and (D). The saliva‐laden swab is held in the sample well of the Anigen Rapid™ FIV PoC test kit and gently blotted while double the recommended test diluent (i.e., 4 drops instead of 2 drops) is dropped onto the cotton tip of the swab (E) and (F), until the fluid begins to move across the test strip. A result is read after 10 minutes, as per the manufacturer's instructions for blood testing (G). The top strip of the test kit is for FIV antibody testing and the bottom strip is for feline leukemia virus (FeLV) antigen testing. One band in the strip indicates a negative result, while two bands in the strip indicates a positive result. In this example, the cat is FIV‐positive with both blood (top kit) and saliva (bottom kit) testing (G). None of the FIV antibody kit manufacturers (including BioNote, manufacturer of Anigen Rapid™) currently endorses using saliva instead of blood as a diagnostic specimen. PoC testing with saliva for the diagnosis of FeLV infection is not recommended. Images provided by Dr Moira van Dorsselaer.

Stress associated with FIV testing should be minimised. Consequently, the use of saliva or ear/pad prick bleeding can be considered as alternatives to venepuncture for FIV PoC testing, even though such testing is “off label” (Figures 5, 6, 7). Saliva testing is discussed in more detail below.

### Point‐of‐care (PoC) FIV testing using whole blood

A variety of FIV PoC antibody test kits are available commercially in Australia and NZ. Clinicians should, whenever possible, use test kits that have been independently validated under local conditions, and exercise caution when using kits that have not been rigorously tested by independent researchers.

**Figure 8:**
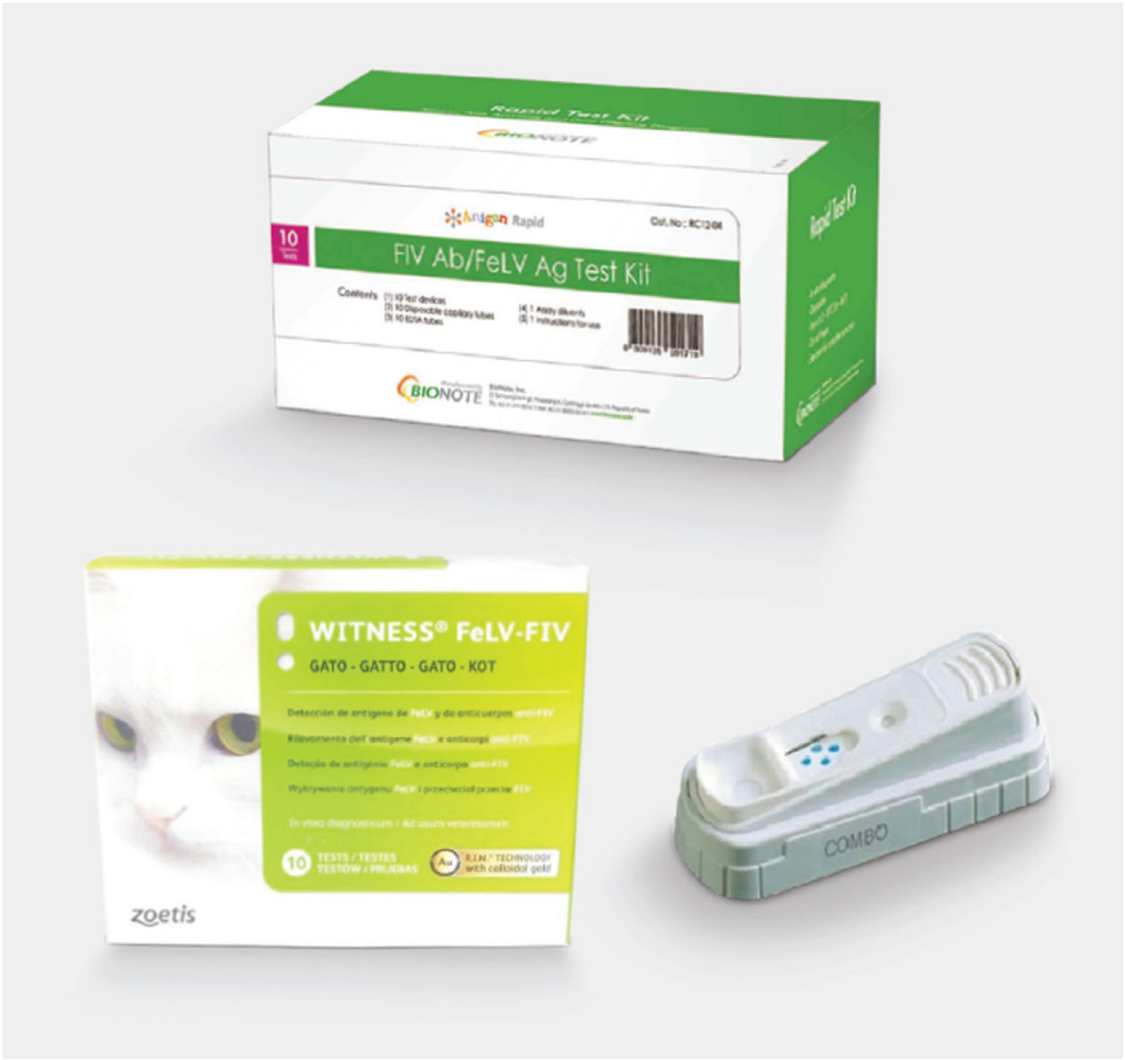
Anigen Rapid™ (top), Witness™ (bottom left) and SNAP Combo™ (bottom right) FIV antibody kits all reliably diagnose infection in FIV‐unvaccinated cats. Anigen Rapid™ and Witness™ kits, however, have an advantage over SNAP Combo™ kits in testing FIV‐vaccinated cats or when FIV vaccination status is unknown as they can differentiate uninfected FIV‐vaccinated and FIV‐infected cats.

In FIV‐unvaccinated cats, three FIV PoC test kits to date have shown good sensitivity (Se) and specificity (Sp) under Australian conditions in an independent study: Anigen Rapid™ (Se 100%, Sp 100%; BioNote, Gyeonggi‐do, Korea), Witness™ (Se 100%, Sp 100%; Zoetis Animal Health, Lyon, France) and SNAP Combo™ (Se 100%, Sp 97%; IDEXX Laboratories, Westbrook, ME, USA) (Figure 8).^67^

In FIV‐vaccinated cats, or when the FIV vaccination status of cats is unknown, the choice of independently validated FIV PoC antibody test kit is more limited. To date, only two test kits, Anigen Rapid™ and Witness™, have demonstrated the ability to distinguish antibodies produced in FIV‐vaccinated cats (cats test negative, unless FIV‐infected) and FIV‐infected cats (infected cats test positive, irrespective of FIV vaccination status). In a cohort of FIV‐vaccinated cats in Australia, the Se/Sp of each kit was 100%/100% (Anigen Rapid™) and 100%/95% (Witness™).^67^ SNAP Combo™ cannot differentiate uninfected FIV‐vaccinated and FIV‐infected cats, with both groups testing FIV‐positive with this kit.^67^, ^68^, ^69^

Anigen Rapid™ and Witness™ FIV kits, however, should be used with caution in kittens and cats that have recently (< 6 months) been given a primary course of three FIV vaccines, with a false‐positive rate of up to 67% reported two weeks after the second primary FIV vaccine dose.^68^ If FIV testing is required during this period, confirmatory FIV PCR testing is recommended in cats that test positive with Anigen Rapid™ or Witness™. Both PoC kits are reliable to use in cats that have recently been given an annual FIV vaccination.^70^ SNAP Combo™ kits should never be used in any FIV‐vaccinated cats, irrespective of how recently a primary or annual FIV vaccination has been administered.

### Point‐of‐care (PoC) FIV testing using saliva

Although testing of whole blood, serum or plasma remains the gold standard for FIV diagnosis, saliva testing for FIV antibodies using Anigen Rapid™ kits may offer a welfare‐friendly alternative to blood testing. In an Australian pilot study, 14 FIV‐infected cats (including 10 FIV‐unvaccinated and 4 FIV‐vaccinated cats) were tested with Anigen Rapid™, Witness™ and SNAP Combo™ PoC kits using saliva instead of whole blood. The Anigen Rapid™ kit performed superiorly to the other kits (14/14 correctly diagnosed as FIV‐infected, i.e., 100% Se).^71^ Anecdotally, limited in‐clinic FIV testing by one of the authors (MvD) with the Anigen Rapid™ kit using both saliva and whole blood has produced comparable results. Validation work for saliva testing using Anigen Rapid™ kits in Australia is currently underway.

Saliva testing in a clinic or shelter setting is often easier to perform than blood testing, and is also less stressful for the cat, its owner and staff member(s) performing the test. In particular, testing with saliva is beneficial in kittens and in shelters where staff need not be trained in venepuncture. Our experience is that although cats resent the cotton swab, they tolerate it. We repeat the saliva collection process a couple of times to ensure that the swab has sufficient sample on it, giving the cat a break between samplings (Figure 7). Conversely, some cats actually tolerate blood sampling better than saliva sampling, due to the unusual sensation of a dry cotton tip on the gums, and in these instances blood sampling should be pursued (e.g., blood collection from the ear tip or foot pad; Figures 5 and 6).

None of the FIV antibody kit manufacturers currently endorses using saliva instead of blood as a diagnostic specimen.

### FIV PCR testing (using blood)

PCR testing detects proviral DNA (FIV DNA inserted into the cat's genome), as well as viral RNA if a reverse‐transcriptase (RT) step is performed as part of the PCR assay. In Australia, the commercially available FIV RealPCR™ (IDEXX Laboratories, East Brisbane, Queensland, Australia) has a published Se and Sp in an independent study of 92% and 99%, respectively,^67^ and includes a RT step to amplify both DNA and RNA in testing. FIV‐infected cats, however, have low levels of both proviral DNA and viral RNA during the long subclinical phase of infection (Figure 3), and some FIV‐infected cats may even enter a stage of lentiviral latency (i.e., undetectable plasma viral replication).^42^, ^72^ Thus, even though PCR testing can detect very low levels of DNA/RNA, it may still fail to detect infection (i.e., produce false‐negative results in FIV‐infected cats).

FIV PCR testing can differentiate uninfected FIV‐vaccinated and FIV‐infected cats (i.e., FIV vaccination does not interfere with the FIV PCR result).

### FIV point‐of‐care (PoC) testing versus FIV PCR testing: How best to approach FIV diagnosis?

FIV antibodies usually rise to detectably high levels within 4–8 weeks of infection and remain high until the clinical phase of FIV infection.^19^, ^41^, ^42^ Even during the clinical phase of FIV infection, when FIV antibody levels may wane, they usually remain high enough to be detected by PoC test kits (Figure 3). Thus, we advise that antibody testing is generally a more reliable method for detecting FIV infection than PCR testing and is our preferred method of FIV diagnosis if possible.

In contrast, FIV PCR testing as a screening or as a confirmatory diagnostic tool in FIV‐unvaccinated cats is not recommended, since its relatively low sensitivity means a negative PCR result does not rule out FIV infection. We are aware of several cases where cats have tested FIV antibody‐positive with PoC kits but have required multiple PCR tests at different time points to confirm FIV infection. It is expensive for the owner, and stressful for the cat, to have multiple samples taken, when testing with a second (and different) PoC antibody kit affords a much faster confirmatory result.

**Figure 9:**
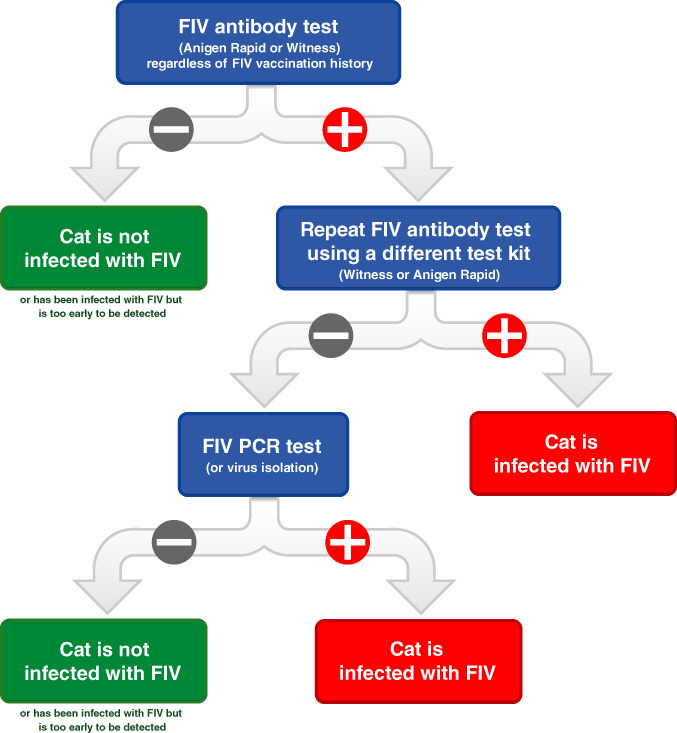
Suggested decision algorithm for diagnosis of FIV infection in FIV‐vaccinated cats and cats with an unknown FIV vaccination history, using blood as the diagnostic specimen. We recommend that cats vaccinated against FIV should undergo annual testing prior to annual FIV re‐vaccination using this algorithm to check infection has not occurred in the preceding year. Taken from Westman et al (2015).^67^

There are, however, some scenarios when FIV PCR testing may be useful to pursue. These include:

- Cats recently administered a primary course of FIV vaccination (< 6 months previously).
- Discordant FIV antibody test results (i.e., one positive and one negative FIV result using different PoC kits; Figure 9).
- Cats recently bitten by another cat when an urgent FIV test result is required (FIV‐infected cats usually test PCR‐positive within 2–4 weeks of being bitten, versus 4–8 weeks for a positive antibody test).^19^, ^41^, ^42^

Tables 1 and 2 provide summaries of general FIV testing recommendations for FIV‐unvaccinated and FIV‐vaccinated cats, respectively, including when PoC saliva testing might be considered. Figure 9 suggests a decision algorithm for FIV testing specifically in FIV‐vaccinated cats and using blood as the diagnostic specimen. It recommends starting with Anigen Rapid™ or Witness™ PoC testing, followed by confirmatory testing with the other kit if a positive result is obtained. If one positive and one negative FIV result is obtained with the two PoC kits, PCR testing is recommended to determine the final FIV status.

**Table 1:** FIV testing recommendations for FIV‐unvaccinated cats in Australia and New Zealand

FIV testing indication	Time of FIV testing	Initial FIV testing	Confirmatory FIV testing
Unwell cat (e.g., unexplained weight loss particularly in a young cat, diarrhoea, lymphadenomegaly, renal disease in a younger cat, behavioural issues, respiratory disease, uveitis, anaemia of unknown cause)	Concurrently with other blood tests (e.g., haematology and biochemistry)	Any independently validated PoC kit (EDTA whole blood)	Use a different independently validated PoC kit (EDTA whole blood)
Blood donation (only enrol cats with no risk of cat fights in previous 12 weeks)	During screening, prior to collecting transfusion blood (will also need blood for FeLV PCR testing)		
Any new kitten or cat (from 12 weeks of age)	At time of initial health check	Any independently validated PoC kit (EDTA whole blood) or Anigen Rapid™ PoC kit (saliva)	Use a different independently validated PoC kit (EDTA whole blood)
Introduction of new cats to a household	Prior to introduction		
Dental procedure	Before dental procedure commences		
Cat fight abscess (CFA)	8 weeks after CFA		
Primary FIV vaccination	At time of health check and prior to first FIV vaccine administration (including recently bitten cats)		

Three commercially available FIV point‐of‐care (PoC) antibody kits to date have been independently validated in Australia in FIV‐unvaccinated cats: Anigen Rapid™, Witness™ and SNAP Combo™. FIV polymerase chain reaction (PCR) testing to detect FIV infection is not recommended since the relatively lower sensitivity compared to PoC testing means that a negative PCR result does not rule out FIV infection. Note that none of the FIV PoC antibody kit manufacturers currently endorses using saliva instead of blood as a diagnostic specimen. FeLV, feline leukaemia virus.

**Table 2:** FIV testing recommendations for FIV‐vaccinated cats and cats with an unknown FIV vaccination history in Australia and New Zealand

FIV testing indication	Time of FIV testing	Initial FIV testing	Confirmatory FIV testing
Unwell cat (e.g., unexplained weight loss particularly in a young cat, diarrhoea, lymphadenomegaly, renal disease in a younger cat, behavioural issues, respiratory disease, uveitis, anaemia of unknown cause)	Concurrently with other blood tests (e.g., haematology and biochemistry)	Anigen Rapid™ or Witness™ PoC kit (EDTA whole blood)	Anigen Rapid™ or Witness™ PoC kit – Whichever was not used for initial testing (EDTA whole blood)
Blood donation (only enrol cats with no risk of cat fights in previous 12 weeks)	During screening, prior to collecting transfusion blood (will also need blood for FeLV PCR testing)		
Annual (or lapsed) FIV vaccination	At time of health check and prior to annual FIV vaccination administration	Anigen Rapid™ PoC kit (EDTA whole blood or saliva) or Witness™ PoC kit (EDTA whole blood)	Anigen Rapid™ or Witness™ PoC kit – Whichever was not used for initial testing (EDTA whole blood)
Cat fight abscess (CFA)	8 weeks after CFA		

Two commercially available FIV point‐of‐care (PoC) antibody kits to date have been independently validated in Australia in FIV‐vaccinated cats: Anigen Rapid™ and Witness™. FIV polymerase chain reaction (PCR) testing to detect FIV infection is not recommended since the relatively lower sensitivity compared to PoC testing means that a negative PCR result does not rule out FIV infection. Note that none of the FIV PoC antibody kit manufacturers currently endorses using saliva instead of blood as a diagnostic specimen. FeLV, feline leukaemia virus.

At the time of writing, IDEXX Laboratories (manufacturer of SNAP Combo™) offers free PCR confirmation of positive FIV SNAP Combo™ test results. This approach is an option for determining FIV status in cats with a history of FIV vaccination or an unknown vaccine history, although PCR results may take several days, and it is important to remember that some FIV‐infected cats may test falsely PCR‐negative during the subclinical phase of infection. In these cases, FIV testing using Anigen Rapid™ and Witness™ PoC kits in series has the advantage of producing rapid, economical and accurate FIV results, and is therefore our recommended approach (Figure 9).

### FIV testing in shelters

It may be useful to know the FIV status of any new kitten or cat upon entry to a shelter to assist with individual management and to limit FIV transmission.^19^ This includes all kittens (>12 weeks of age) and cats rehomed from pounds, shelters, and other rescue facilities. All animal holding facilities should aim to individually house adult cats to limit the spread of FIV infection through fighting. If group housing of cats is practised, the FIV status of all those co‐housed should be determined, so that FIV‐infected cats can be housed individually, and FIV‐infected cats ideally placed into indoor‐only single cat households.^73^ FIV testing of healthy cats going to single cat households may be futile and unnecessary. If shelters opt not to test animals for FIV prior to adoption, new owners should be made aware and a waiver signed to indicate that they understand that the cat's FIV infection status is unknown.

Identifying FIV‐infected cats can affect length‐of‐stay for those animals, which is detrimental to their overall health and welfare, and some argue that not knowing FIV infection status is better for these animals.^74^ In contrast, one study of cats rehomed from a shelter in Canberra, Australia, did not find any difference with regards to the length‐of‐stay of FIV‐infected versus FIV‐uninfected cats.^75^ Many FIV‐infected cats will live normal, happy, healthy lives with appropriate management, and many people will willingly adopt FIV‐infected cats. Alarmingly, 4/17 Australian shelters responding to a questionnaire indicated that they euthanised all FIV‐positive cats regardless of health status.^76^ Euthanasia of healthy FIV‐infected cats because of their FIV status should not occur.^19^, ^41^, ^73^

Shelters are usually rehoming kittens and young healthy animals, often with an unknown FIV vaccination history, and therefore the following key recommendations should be followed where possible:

- FIV antibody testing using PoC kits is more reliable and cheaper than FIV PCR testing and should be used for both initial disease screening and confirmation of any positive FIV test results (using a different manufacturer's test kit) in FIV‐unvaccinated cats (Figure 8).
- Anigen Rapid™ and Witness™ kits (not SNAP Combo™) accurately detect FIV infection in FIV‐vaccinated cats (excluding cats who have recently received a primary vaccination course), and therefore are ideal to use in cats with a history of FIV vaccination or unknown vaccination history (Figure 8).
- If a cat tests FIV‐positive with an Anigen Rapid™ or Witness™ PoC kit, the result should be confirmed with the other FIV test kit (i.e., ideally shelters should stock both kits; Figure 9).
- For some cats, collection of saliva for FIV testing with an Anigen Rapid™ kit reduces sampling stress and should be pursued instead of blood collection (Figure 7). Results from an Australian pilot study suggest that if a cat tests FIV‐negative with Anigen Rapid™ using saliva, further blood testing is not required.^71^
- If blood collection is needed (e.g., for confirmatory FIV testing with Witness™ after a positive Anigen Rapid™ result using saliva), foot pad bleeding or ear tip bleeding are low stress options for some cats (Figures 5 and 6).
- Kittens can be tested for FIV from 12 weeks. This is younger than most major retroviral guidelines suggest. In one study involving 55 kittens born to 12 uninfected FIV‐vaccinated queens, 30/55 (55%) kittens tested FIV‐positive with SNAP Combo™ at 8 weeks of age, but all 55 kittens tested FIV‐negative by 12 weeks of age, demonstrating the decay of maternally‐derived antibodies (MDA) from FIV vaccination by this age.^77^ Some Australian shelters now perform FIV testing in litters of kittens less than 12 weeks old; in this scenario, negative FIV results can be trusted, but positive FIV results must be confirmed by either PCR testing or repeat PoC testing using blood when the kitten is older (e.g., re‐test in 1 month).
- FIV testing should be performed on all animals prior to dental procedures. Freshly autoclaved dental instruments should be used for each cat, irrespective of the FIV result (in case of early FIV infection prior to seroconversion). Thorough cleaning of all non‐autoclavable equipment should be undertaken at the conclusion of every dental procedure.^19^
- Spays and castrations should never be done using a single kit for multiple cats. Sterile, autoclaved surgical instruments should be used for each new procedure.^19^

## Prevention of FIV infection

### Keeping cats indoors

Keeping pet cats indoors, including secure outdoor enclosures, is the cornerstone of FIV prevention and is the best way of ensuring longevity for owned cats. It will also help prevent vehicular trauma, UV‐induced skin cancer, snake bites and tick paralysis, while simultaneously minimising adverse impacts of pet cats on wildlife.^78^, ^79^

Bite wounds inflicted by FIV‐infected cats have the potential to transmit FIV from one cat to the next.^12^ Such bite wounds are most likely to occur in pet cats that are allowed to go outdoors, unsupervised.^14^, ^15^, ^16^, ^30^, ^31^, ^80^ Indoor pet cats may occasionally squabble within their social groups but are less likely to inflict bite wounds on one another. Even if the FIV status of all cats in a multi‐cat household is not yet known, FIV transmission is relatively uncommon in stable, established groups kept indoors.^81^

If one or more cats in a multi‐cat household becomes ill and is discovered to be FIV‐infected, it is advisable to test the remaining cats.^19^ Whether or not to advise separation of infected from uninfected cats depends on whether there is a history of inter‐cat aggression and wounding within the indoor social group.

**Figure 10:**
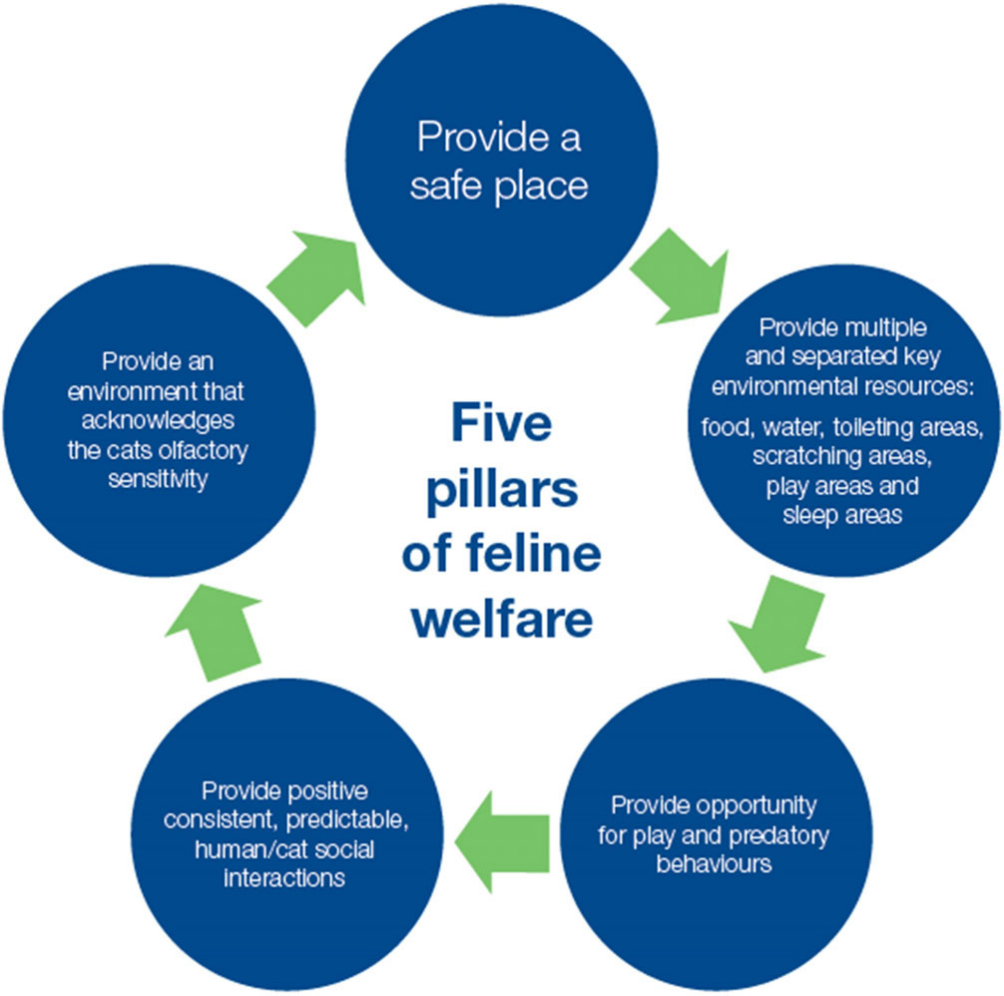
Keeping cats housed 100% indoors happy requires fulfilling each of the five “pillars” for a healthy feline environment. Adapted from the 2013 American Association of Feline Practitioners (AAFP) and International Society of Feline Medicine (ISFM) feline environmental needs guidelines.^84^

Meeting the welfare needs of cats kept 100% indoors is vital. This topic has been studied increasingly in recent years.^82^, ^83^, ^84^, ^85^, ^86^ Familiarity with the American Association of Feline Practitioners (AAFP) and International Society of Feline Medicine (ISFM) Feline Environmental Needs Guidelines may help when advising and encouraging clients weighing up whether or not to keep their pet cat(s) 100% indoors, or indoors with access to an outdoor secured enclosure. Their “Five Pillars for a Healthy Feline Environment” are helpful (Figure 10).^84^

Useful advice about play, exercise, weight control, environmental enrichment and avoidance of separation anxiety has been gathered and presented as part of the “Indoor Pet Initiative.”^87^ This resource provides an abundance of encouragement and advice about refuges, prey‐preference‐selected toys, perches, resting areas and much more for veterinarians and cat owners. Indoor‐outdoor secured enclosures can be home‐made, or purpose designed. They can be lavish and extensive.

Not all clients can manage to house their cat(s) 100% indoors, while managing to completely fulfill their welfare requirements.^36^ Consequently, FIV vaccination may need to be considered, depending on individual risk factors for FIV infection (e.g., local FIV prevalence in owned and unowned cats, density of pet cats in the local area with outdoor access, size of the unowned cat population, etc.).^88^

### FIV vaccination

Fel‐O‐Vax® FIV (distributed by Boehringer Ingelheim Animal Health in Australia and Zoetis in NZ) is an inactivated, whole‐virus vaccine that contains two divergent subtypes (A and D) of the virus, thought to broaden the protection it affords. The efficacy of this vaccine has been studied by multiple research groups over the last 20 years. Different methodologies and challenge doses and strains were used in these studies.

Widely varying results have been obtained. Several studies have demonstrated 100% protection,^5^, ^89^, ^90^, ^91^, ^92^, ^93^, ^94^ while one small study showed no efficacy at all.^95^ An Australian case–control field study published in 2016 showed a vaccine protective rate of 56%.^9^ Stringent inclusion criteria were applied in this study, reducing the number of cases and controls, leaving the study statistically underpowered. Nevertheless, this provided sufficient evidence for the World Small Animal Veterinary Association's (WSAVA) Vaccination Guidelines Group to redesignate this vaccine as “Non‐Core”; whereas it had previously been designated as “Not Recommended.”^88^ “Non‐Core” vaccines can be recommended after careful risk–benefit analysis has been performed by the veterinarian and pet owner in consultation.

A NZ field study recently reported no protection from FIV infection in FIV‐vaccinated cats.^11^ The definition for FIV‐positivity was more relaxed in the NZ study and there were biases with case recruitment that impacted study results. For example, the rate of FIV infection in controls was half that of vaccinates, suggesting differences between the recruited groups. Despite this, the result is important and might reflect differences in circulating NZ subtypes of FIV and resulting impact on vaccine efficacy. Further field investigations of FIV vaccine efficacy clearly should be undertaken to explore these differences.

FIV vaccination can be recommended for pet cats whose owners are unable to, or cannot be persuaded to, keep their cats away from the risk of being bitten by an FIV‐infected cat. Unfortunately, many cats fall into this category because a majority of cats in Australia and NZ are allowed to go outdoors on their own, unsupervised, and FIV infection is prevalent in such cats across both countries.^14^, ^25^, ^26^, ^67^, ^96^

If a kitten/cat does not receive the full set of three primary Fel‐O‐Vax® FIV vaccine doses on schedule (administered 2–4 weeks apart), or an adult cat is late for its annual FIV booster, seek advice from the vaccine manufacturer as to the optimal vaccination regimen.

There is no known benefit to administering Fel‐O‐Vax® FIV to an already FIV‐infected cat. Therefore, kittens and cats should be determined to be FIV‐uninfected prior to commencing FIV vaccination by FIV PoC antibody testing (Table 1). The manufacturer of Fel‐O‐Vax® FIV currently only recommends FIV testing kittens >6 months of age pre‐vaccination, despite FIV PoC testing in kittens being reliable from 12 weeks of age.^77^ We advise veterinarians to use their clinical judgement and also test kittens between 3 and 6 months of age if they have been at‐risk of FIV transmission (e.g., some level of unsupervised outdoor access, presence of cat fight wounds, or born to a FIV‐infected queen). In addition, since Fel‐O‐Vax® FIV has reports in Australia and NZ of field effectiveness less than 100%, we recommend that cats vaccinated against FIV should undergo annual testing prior to annual FIV re‐vaccination to check infection has not occurred in the preceding year, using an Anigen Rapid™ or Witness™ PoC antibody kit (Table 2, Figure 9).^9^, ^11^

### Suggested FIV vaccination procedure for recently bitten cats

Since FIV is most commonly transmitted during fighting, it is the cats that constantly get into fights and being bitten (“brawlers”) who are at the greatest risk of becoming FIV‐infected.^12^, ^15^, ^30^, ^31^ It is these cats who have the most to benefit from FIV vaccination. It can be hard to know when to vaccinate these cats, as some owners will not bring their cat back to the clinic for testing. Waiting eight weeks to use a PoC antibody test kit might result in another cat fight bite in the intervening period, with the 8‐week countdown for antibody testing re‐starting.

For this reason, we suggest vaccinating "brawlers" against FIV at the first possible opportunity (even if recently bitten), using the following protocol and as soon as the cat fight injury has resolved:

- Perform FIV testing with an independently validated PoC kit to ensure the cat was not previously FIV‐infected (note, however, that this may not detect infection caused by the most recent fight); administer the first primary Fel‐O‐Vax® FIV dose.
- 2–4 weeks later: administer the second primary Fel‐O‐Vax® FIV dose.
- 2–4 weeks later: administer the third primary Fel‐O‐Vax® FIV dose.
- 12 months later: FIV testing with an Anigen Rapid™ or Witness™ PoC kit immediately prior to administration of the first annual Fel‐O‐Vax® FIV vaccine (Table 2, Figure 9). If FIV‐infected, cease FIV vaccination.

If a "brawler" cat tests FIV‐positive at the first annual FIV vaccination, it will be impossible to determine if the cat became FIV‐infected prior to or during the completion of the primary FIV vaccination course, or if FIV infection became established following primary FIV vaccination and before annual FIV re‐vaccination. Consequently, reporting these cases as lack of efficacy events (“vaccine failures”) for the Fel‐O‐Vax® FIV vaccine would be inappropriate.

### Feline injection‐site sarcomas (FISS)

The most serious risk of vaccination (and other injections) in cats is feline injection‐site sarcoma (FISS) formation. This is a very rare, malignant neoplasm thought to be attributable to injection of vaccines and other substances.^97^, ^98^ It was first described in the USA in 1991 and reported shortly afterwards in Australia.^99^ The incidence of FISS is thought to be much lower in Australia and NZ than the USA, in part due to absence of rabies vaccination, but good quality local epidemiological data are lacking. Nevertheless, the consequences for affected cats are catastrophic. Given the apparent rarity of these tumours in Australia and NZ, we do not currently encourage distal limb or tail vaccination. However, avoiding the interscapular furrow, and injecting approximately 4 cm lateral to the dorsal midline, over the convexity of the muscles covering the scapula spine, would allow earlier detection and diagnosis of any mass subsequent to vaccination. Although the role of adjuvants in the pathogenesis of FISS is unresolved, we suggest varying the site of injection of adjuvanted vaccines from year to year (i.e., left side one year, right side the next). The site of injection of each vaccine should be recorded in the medical record.^98^

## Management of FIV‐infected cats

Critically, the term “Feline AIDS” (“FAIDS”) to describe FIV‐infected cats is outdated and should not be used. Whilst many owners will try to make comparisons between the clinical course, therapeutic interventions, and outcomes for HIV and FIV infections, key differences exist. Importantly, most FIV‐infected cats reach reasonable longevity without antiretroviral drugs when there is diligent attention to husbandry and management of concurrent disease.^16^, ^30^, ^62^, ^64^, ^100^ The best way to enhance quality of life and life expectancy in FIV‐infected cats is to optimise basic husbandry and to detect and treat any concurrent conditions early in the disease course.

It is important to stress to owners that FIV is not a death sentence and does not constitute grounds for euthanasia.^19^, ^41^

**Figure 11:**
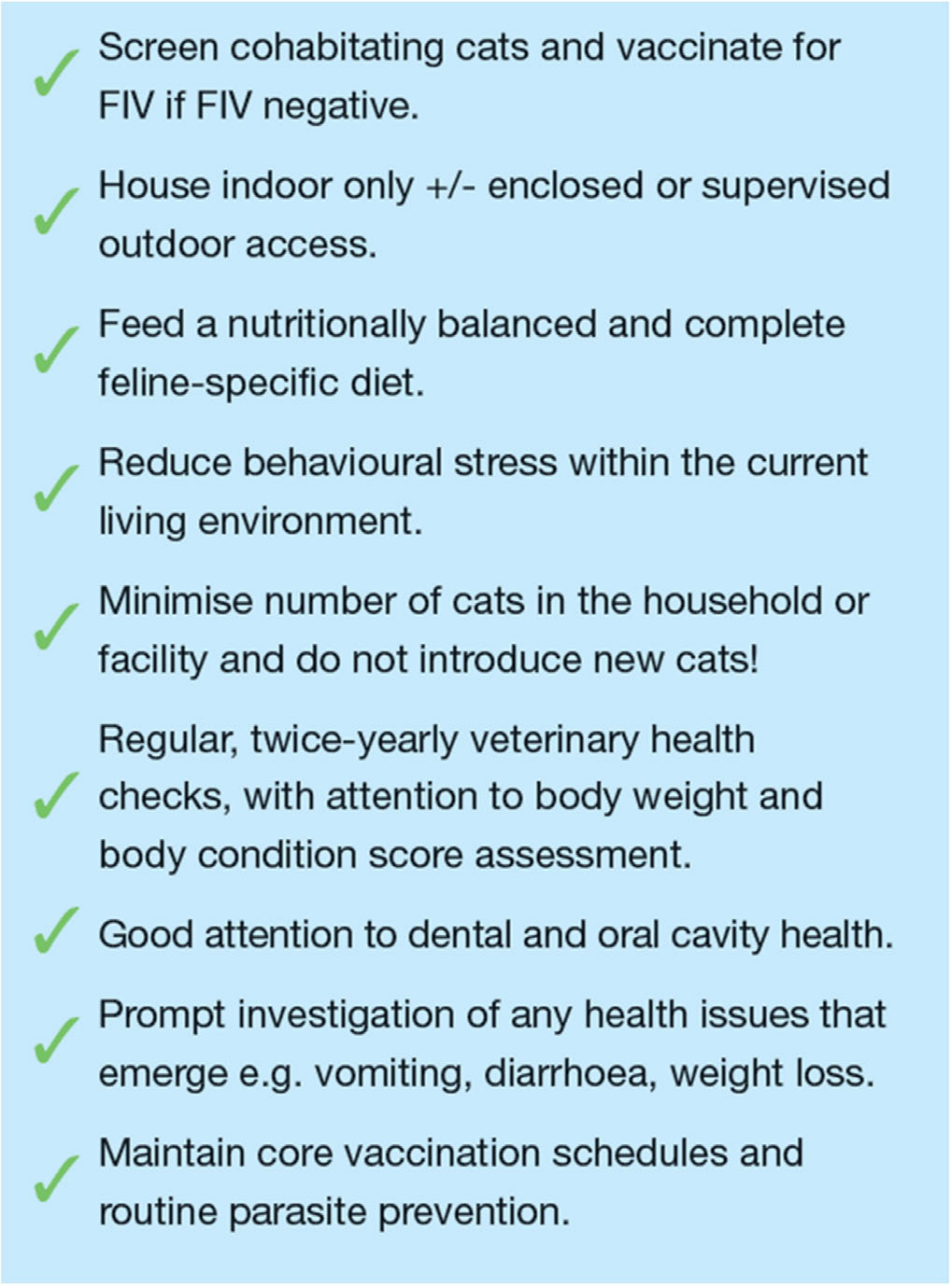
A suggested checklist of recommendations for basic health care and husbandry measures for all FIV‐infected cats.

When approaching management of FIV‐infected cats, the guiding principles are:

- Clinicians should strive to be even more thorough than usual at detecting early signs of disease and err on the side of “sooner rather than later” so that possible diseases can be diagnosed and treated in a timely manner. For example, managing early periodontal disease by regular and assiduous scaling, polishing and extraction of teeth before more severe changes can develop. Likewise, even mild flea infestations should be managed with modern effective monthly parasiticides (see Figure 11 for a checklist of health recommendations).
- Owners should be encouraged to take an “if in doubt get it checked out” approach and act as soon as any concerning clinical signs or behaviours are noted.
- Diseases that would be considered possible or likely in cats without FIV infection should be excluded via thorough clinical investigation before ascribing any clinical problem to the virus itself.

### Monitoring for immunodeficiency

A complete blood count can be performed on FIV‐infected cats at the time of diagnosis and annually thereafter to monitor for haematologic abnormalities such as leukopenia and anaemia.^49^, ^59^, ^98^, ^101^

The presence of lymphopenia in particular may signal declining immune function, but it is a common finding in stressed sick cats.^65^, ^101^ Cat owners may be aware of the use of CD4+ T lymphocyte, plasma viral RNA load, and acute phase protein quantification to monitor immune status and disease progression in people infected with HIV. Whilst monitoring of equivalent measurements has been reported for cats naturally infected with FIV,^65^, ^102^, ^103^ it is not currently utilised widely by clinical veterinarians. Cats can live with low CD4+ T lymphocyte counts for prolonged periods without developing problems, a fact that remains an enigma to feline immunologists.^19^ At the time of writing, quantification of lymphocyte subsets by flow cytometry (CD4+ and CD8+ numbers, CD4+/CD8+ ratio and a panleukocyte marker for gating purposes) is commercially available in Australia and costs $190.00 AUD (Vetnostics and partner laboratories QML and ASAP). Perhaps more flow cytometry should be done to stage FIV‐infected cats.

Measurement of plasma viral RNA load and concentrations of acute phase proteins such as serum amyloid A and C‐reactive protein may prove to be useful in disease staging and prognostication for FIV‐infected cats in the future,^103^ but criteria are yet to be defined.

### Antiretroviral and immunomodulatory agents: Which drugs might clinicians consider?

Antiretroviral drugs are not currently indicated for most FIV‐infected cats, with any decision to implement antiretroviral treatment comprising a risk–benefit decision that must be made on a case‐by case basis. At this stage any drug treatment of FIV infection is best considered experimental, and the authors are unaware of feline clinicians in Australia or NZ who currently use specific antiretroviral therapy to manage cats with FIV infections. None of the drugs discussed below are registered for the treatment of FIV‐infected cats.

Discussion of drug therapy must reflect existing survival data for infected cats as well as the health status of the individual cat. With regard to specific antiviral treatment for FIV, major barriers to potential success include limited drug availability and absent field trial data, frequent toxic side‐effects, high cost, impractical administration frequency and duration of therapy. A decision to treat remains a risk–benefit decision to be made on a case‐by‐case basis, and in many instances, antiretroviral viral therapy may present more risks than benefits.^104^

Of the antiretroviral drugs investigated, zidovudine (also known as azidothymidine; AZT), a nucleoside analogue reverse transcriptase inhibitor, holds the most promise for use in FIV‐infected cats. AZT has been shown to reduce viral load, with improvements in immunologic status (including CD4+/CD8+ ratios), quality of life, life‐expectancy and clinical status, particularly in neurologic presentations of FIV or FIV‐associated stomatitis.^104^, ^105^, ^106^, ^107^ Drug resistance has been reported, as has bone marrow suppression, primarily manifesting as non‐regenerative anaemia. AZT is consequently not recommended for use in cats with evidence of pre‐existing bone marrow suppression.^104^ Vomiting and anorexia have been reported but occur infrequently and the drug otherwise appears to be well tolerated. A regimen comprising 5–10 mg/kg PO q12h with weekly monitoring of haematology for the first month, then monthly monitoring thereafter, may be recommended.^106^, ^107^

Interferons (human and feline) mediate and enhance innate immunity as a means of reducing susceptibility to secondary infections and viral replication.^102^ Well‐designed clinical trials are lacking, although a recent Spanish study reported that oral administration of human interferon alpha for a period of four months improved haematologic parameters (including the CD4+/CD8+ ratio) and was well tolerated by naturally FIV‐infected cats.^108^ Feline interferon omega (Virbagen Omega, Virbac Animal Health, Australia) has also been utilised and is well tolerated. A small number of trials have shown evidence of clinical improvement in FIV‐infected cats, although its true therapeutic effect requires further demonstration.^109^, ^110^

## Conclusion and future priorities

Much has changed with regards to the diagnosis of FIV and approach to the treatment and management of FIV‐infected cats. This review updates Australian and NZ clinicians on these recent advances and summarises current knowledge and approach to FIV infection. More practical research investigating the impact of FIV infection on health and longevity, and FIV vaccine effectiveness in the field, is desperately needed in both Australia and NZ.

## Conflicts of interest and sources of funding

Boehringer Ingelheim Animal Health Australia, supplier of the Fel‐O‐Vax® FIV vaccine in Australia, provided an honorarium to all listed authors to create a documented titled “Australian Feline Retrovirus Management Guidelines: Part 1 Feline Immunodeficiency Virus (FIV).” This document heavily informed the writing of this manuscript. Boehringer Ingelheim Animal Health Australia did not ask the authors to publish this document in a peer‐reviewed journal and was entirely an independent decision of the authors listed. The authors declare no sources of funding for the work presented here.
